# Association of Gut Dysbiosis with Disease Phenotype and Treatment in Systemic Lupus Erythematosus

**DOI:** 10.3390/medsci13030151

**Published:** 2025-08-23

**Authors:** Irene Medina-Martínez, Rocío Gil-Gutiérrez, Jorge García-García, Francisco Javier de la Hera-Fernández, Nuria Navarrete-Navarrete, Mónica Zamora-Pasadas, Norberto Ortego-Centeno, José Luis Callejas-Rubio, Federico García-García, Julio Gálvez-Peralta, Alba Rodríguez-Nogales, María Correa-Rodríguez, Blanca Rueda-Medina

**Affiliations:** 1 University of Granada, 18016 Granada, Spain; irene.medina.mrtnz@gmail.com; 2 Department of Nursing, Faculty of Health Sciences, University of Granada, 18071 Granada, Spain; rogilgu@ugr.es (R.G.-G.); macoro@ugr.es (M.C.-R.); blarume@ugr.es (B.R.-M.); 3 Institute for Biosanitary Research of Granada (ibs.Granada), 18012 Granada, Spain; javijanjan@hotmail.com (F.J.d.l.H.-F.); nortego@ugr.es (N.O.-C.); jlcalleja@ugr.es (J.L.C.-R.); fegarcia@ugr.es (F.G.-G.); 4 Microbiology Service, San Cecilio University Hospital, 18016 Granada, Spain; 5 Systemic Autoimmune Diseases Unit, San Cecilio University Hospital, 18016 Granada, Spain; 6 Systemic Autoimmune Diseases Unit, Virgen de las Nieves University Hospital, 18014 Granada, Spain; nurianavarreten@ugr.es (N.N.-N.); monikazamora@hotmail.com (M.Z.-P.); 7 Department of Medicine, Faculty of Medicine, University of Granada, 18071 Granada, Spain; 8 Department of Clinical Microbiology, San Cecilio University Hospital, 18016 Granada, Spain; 9 Biomedical Research Network Center, Infectious Diseases (CIBER-INFEC), 18100 Granada, Spain; 10 Department of Pharmacology, Center for Biomedical Research (CIBM), University of Granada, 18071 Granada, Spain; jgalvez@ugr.es; 11 Biomedical Research Network Center, Liver and Digestive Diseases (CIBER-EHD), 18016 Granada, Spain

**Keywords:** systemic lupus erythematosus, gastrointestinal microbiome, dysbiosis, metagenomic sequencing, autoimmune diseases, hypertension, drug therapy

## Abstract

**Introduction**: Gut dysbiosis has been associated with the development of autoimmune diseases, including systemic lupus erythematosus (SLE). Although previous studies suggest microbial alterations in SLE, evidence at the species level and its clinical relevance remain limited. This study aimed to characterise the gut microbiota at species level in SLE patients and evaluate its association with clinical features. **Materials and methods**: A total of 57 SLE patients and 57 matched controls were included. Faecal samples were collected using the OMNIgene-GUT kit, and microbial DNA was extracted with the Maxwell RSC PureFood GMO kit. Metagenomic sequencing was performed using the Illumina MiSeq platform, and the data was analysed with QIIME2. Microbial diversity and relative abundance were assessed using the phyloseq package, and differentially abundant taxa were identified using DESeq2. Clinical subgroups among SLE patients were identified via k-means clustering. **Results**: SLE patients exhibited significantly different beta diversity compared to controls (*p* = 0.001), with increased abundance of *Pseudomonadota* (3.81% vs. 6.80%, *p* < 0.05) and decreased *Bacteroidota* (53.42% vs. 38.04%, *p* < 0.05). Only 10 bacterial species were consistently present across all SLE samples, including *Akkermansia muciniphila*, *Bacteroides dorei*, and *Lactobacillus gasseri.* Hypertensive patients and those treated with corticosteroids presented a marked depletion of key microbial taxa. Conversely, Belimumab-treated patients displayed a distinct microbiota enriched in species such as *Alistipes shahii* and *Prevotella corporis*. **Conclusions**: This study confirms significant gut microbiota alterations in SLE and pinpoints microbial profiles associated with clinical subgroups. These findings suggest gut dysbiosis may contribute to SLE pathogenesis and indicate biomarkers for disease stratification.

## 1. Introduction

Recently, the gut microbiome has emerged as a critical modulator of immune function and a potential contributor to the pathogenesis of autoimmune diseases such as rheumatoid arthritis, Sjögren’s syndrome, multiple sclerosis, autoimmune thyroid disease, type 1 diabetes mellitus, ulcerative colitis, psoriasis, coeliac disease, and systemic lupus erythematosus (SLE). It plays a central role in maintaining immune homeostasis and shaping mucosal and systemic immune responses [1]. Disruption of this microbial balance (known as dysbiosis) has increasingly been shown to be involved in the initiation and progression of autoimmunity [2]. In the context of SLE, dysbiosis may contribute immune dysfunction through mechanisms such as increased intestinal permeability (known as “leaky gut”), molecular mimicry, epigenetic modifications, and synergistic interactions with host genetics, environmental exposure, and microbial communities [3].

Leaky gut, characterised by compromised intestinal barrier integrity, facilitates the translocation of microbial products and antigens into the bloodstream, potentially triggering aberrant immune activation and sustained inflammation. Molecular mimicry, where microbial antigens resemble host tissues, may activate autoreactive T and B cells, leading to the production of pathogenic autoantibodies [4]. Additionally, the gut microbiota can modulate epigenetic landscapes, altering gene expression through DNA methylation and histone modification, ultimately impacting immune cell function and disease expression [4].

This intricate triad of microbiota, genetics, and environment adds another layer of complexity to SLE pathogenesis. Recent studies have identified microbial signatures correlated with disease activity and flare frequency, offering potential biomarkers for disease monitoring and stratification [5,6]. SLE patients often exhibit reduced levels of immunoregulatory bacteria such as *Lactobacillus*, *Bifidobacterium*, and *Akkermansia muciniphila*, alongside an enrichment of pro-inflammatory taxa including *Fusobacterium* and *Prevotella* [4]. These microbial shifts may contribute to the persistent inflammation and immune dysregulation that characterise SLE.

However, despite the growing body of evidence linking gut dysbiosis to systemic SLE, the relationship between microbial alterations at the species level and disease-associated clinical phenotypes (such as hypertension and cardiovascular risk) remains insufficiently understood. Clarifying these associations is crucial, as identifying potential microbiome-based biomarkers related to disease severity and clinical subtypes could pave the way for precision medicine strategies tailored to individual patient profiles. This could include evaluating treatment effectiveness based on microbiome changes or incorporating specific probiotics into disease management.

In this study, we aimed to characterise the gut microbiome of SLE patients at the species level and explore its association with specific clinical manifestations. By identifying microbial signatures linked to disease activity and phenotypic diversity, we hope to advance the development of targeted, microbiome-informed therapies and improve treatment outcomes for individuals with SLE.

## 2. Materials and Methods

### 2.1. Study Design and Population

This observational study included 57 female patients with systemic lupus erythematosus (SLE), recruited from San Cecilio and Virgen de las Nieves University Hospitals in Granada, Spain. All the participants provided informed consent, and the study was approved by the Biomedical Research Ethics Committee of Granada (2099-N-21).

Eligibility required a confirmed SLE diagnosis at least one year prior, based on criteria from the American College of Rheumatology (ACR), European League Against Rheumatism (EULAR), or Systemic Lupus International Collaborating Clinics (SLICC). The diagnostic criteria were established based on the presence of certain clinical manifestations such as fever, malar rash, discoid rash, photosensitivity, oral ulcers, arthritis, serositis, renal disease, haematological, neurological, and/or immunological disorders. The participants had to have stable SLEDAI-2K (Systemic Lupus Erythematosus Disease Activity Index 2000) scores and no treatment changes in the preceding three months.

Additionally, medium (8–11 points) or high (12–14 points) adherence to the Mediterranean Diet, assessed using the PREDIMED study scale, was required.

The exclusion criteria included terminal illness, serum creatinine ≥ 1.5 mg/dL, type 1 diabetes, recent trauma or surgery (within six months), pregnancy, breastfeeding, or other autoimmune/inflammatory diseases.

As a control group, 57 healthy women matched by age and body mass index (BMI) were included.

### 2.2. Faecal Sample Collection and DNA Isolation

Participants collected faecal samples at home using the OMNIgene·GUT kit (DNAgenotek; Ottawa, Canada), which were stored frozen (−80 °C) until analysis (24 weeks). Microbial DNA was extracted using the Maxwell RSC PureFood GMO and Authentication kit (Promega; Madison, WI, USA), following the manufacturer’s protocol.

### 2.3. Metagenomic Sequencing, Quality Control, and Taxonomic Assignment

The DNA was extracted according to the method by Rodríguez-Nogales et al. [7]. Total DNA was amplified using primers targeting the V3–V4 regions of the bacterial 16S rRNA gene. PCR products were purified via gel electrophoresis, analysed using multiplexing on the Illumina MiSeq platform (Illumina Inc., San Diego, CA, USA), and verified using high-throughput Invitrogen 96-well E-gel (Thermo Fisher Scientific, Waltham, MA, USA). The samples were subsequently pooled, cleaned, and standardised with the SequalPrep 96-well Plate kit. Libraries were fluorometrically quantified before sequencing. Next-generation sequencing (NGS) was performed using the Illumina MiSeq platform, and the raw data was used for the microbiome composition analysis.

### 2.4. Bioinformatics and Statistical Analyses

Bioinformatic processing was conducted using the QIIME2 pipeline (Northern Arizona University, Flagstaff, AZ, USA) [8]. The sequences were trimmed and filtered based on quality scores [9]. DADA2 was used for denoising, and amplicon sequence variants (ASVs) were generated. Taxonomic classification was performed using the SILVA database [10], excluding Archaea and Eukaryota.

Statistical analyses were run using R software (version 4.3.2) [11]. Clinical variables were expressed as the mean ± the standard deviation (SD) or the median and interquartile range, depending on the distribution. Categorical variables were presented as percentages. The microbiota composition was analysed using the phyloseq package to assess alpha and beta diversity and relative abundance. The *t*-test or Wilcoxon test was used to evaluate significant differences depending on the data distribution, which was assessed using the Shapiro–Wilk test. Beta diversity differences were analysed using permutational multivariate analysis of variance (PERMANOVA) with the Adonis function.

Visualisations such as Venn diagrams, heatmaps, and correlation plots were generated using the Eulerr and MicroViz packages. Pearson correlation coefficients were used for correlation analysis. Differential taxa expression was assessed using the DESeq2 package (version 4.2), and potential biomarkers were identified using linear discriminant analysis (LDA) effect size (LEfSe), with an LDA score threshold of 3.

Lastly, clusters based on clinical variables from SLE patients were generated using machine learning with the k-means method. Categorical variables were converted into dummy variables using the caret package, and numerical variables were scaled for comparability. The optimal number of clusters was determined using the elbow method based on within-cluster sum of squares (WSS), implemented via the factoextra package. K-means clustering was run with 25 initialisations to ensure stability. Principal component analysis (PCA) was used to evaluate the contribution of each variable to the clustering.

## 3. Results

### 3.1. Participant Characteristics and Clinical Outcome

The main characteristics of the study groups are shown in Table 1. The study participants had a mean age of 44.19 ± 11.88 years, mean weight of 61.83 ± 12.65 kg, and a mean BMI of 23.78 ± 4.75, with no significant differences compared to the control group, as was expected (Table 1). The mean time since diagnosis was 11.07 ± 8.87 years, with a mean SLEDAI-2K of 5.81 ± 5.91 and a mean SLICC of 1.05 ± 1.33. Among the SLE patients, 40.35% were taking corticosteroids, and the most common treatment was antimalarial (87.71%). The patients were being treated with a mean of 3.12 ± 1.98 drugs. The most common comorbidities in the SLE group were hypertension (28.07%), arthritis (24.56%), kidney disease (17.54%), and osteopenia (14.03%).

### 3.2. Alterations in the Gut Microbiota Composition of SLE Patients

**Figure 1:**
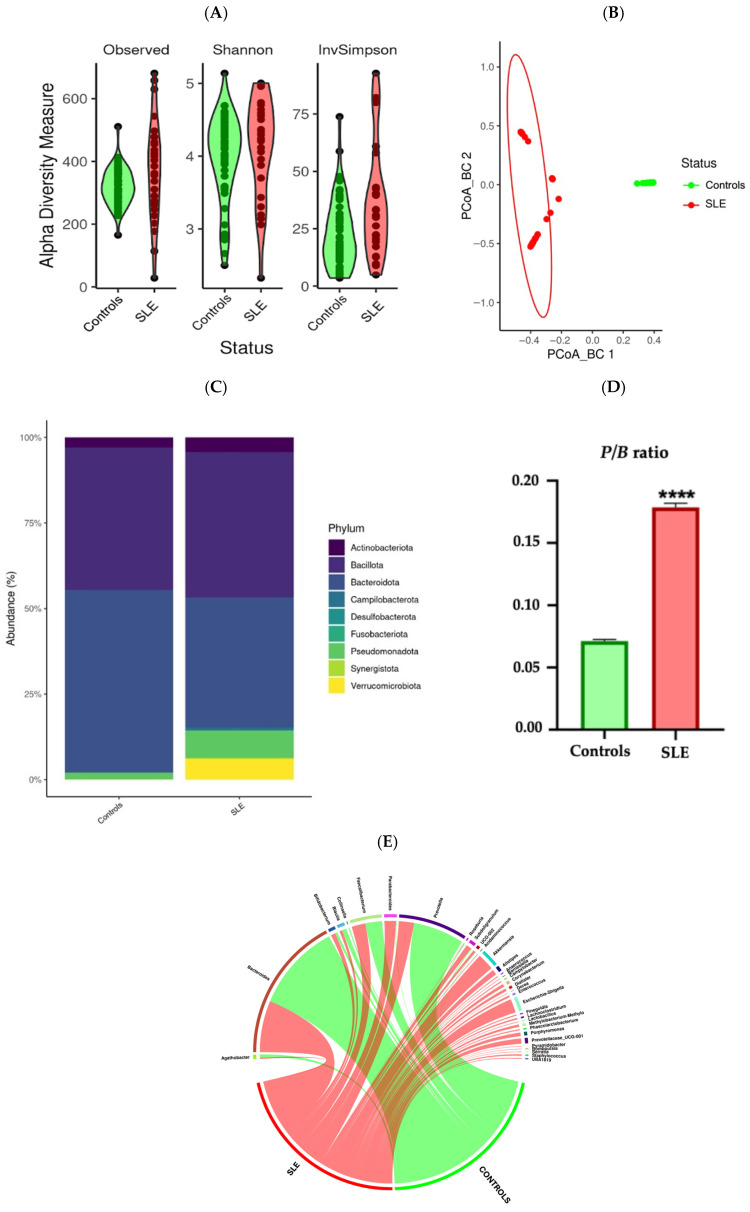
Gut microbiota diversity at phylum and genus level in SLE patients and controls. The SLE patients showed diversity differences in their gut microbiota. (**A**) Alpha diversity (observed features, Shannon, and inverse Simpson) between the control and SLE groups. (**B**) PCoA for Bray–Curtis diversity comparing the control and SLE samples. (**C**) Differences between SLE patients and controls at phylum level. (**D**) Altered *Pseudomonadota/Bacteroidota* (*P*/*B*) ratio in SLE patients. (**E**) SLE patients and controls presented differences in certain taxa at genus level. **** stands for a *p* value < 0.001.

To compare gut microbiota diversity between SLE patients and healthy controls, alpha and beta diversity analyses were conducted (Figure 1). Alpha diversity analysis revealed no significant differences between the groups, suggesting similar microbial richness and evenness, as measured by the Shannon index (richness) and the inverse Simpson index (evenness) (Figure 1A). Shannon index values were comparable (*p* > 0.05), indicating that the number of distinct microbial species was relatively consistent across the groups, and no significant variation in the inverse Simpson index was observed (*p* = 0.06), suggesting that the overall microbial diversity was preserved across the groups.

In contrast, the beta diversity analysis revealed significant structural differences in the microbiome composition. Principal coordinate analysis (PCoA) using the Bray–Curtis dissimilarity showed a clear separation between the two groups (PCoA Bray–Curtis *p* = 0.001) (Figure 1B).

At the phylum level (Figure 1C), the predominant taxa across the two groups were *Bacteroidota* and *Bacillota*. However, the SLE patients exhibited a notable shift in microbial composition compared to the controls, characterised by a significant increase in *Pseudomonadota* (*P*) (3.81% vs. 6.80% *p* < 0.05) and decrease in *Bacteroidota* (*B*) (from 53.42% to 38.04%, *p* < 0.05) (Figure 1C). No significant differences in the distribution of the phylum *Bacillota* were found between the controls and the SLE patients (from 41.62% to 42.35%, *p* > 0.05), but we did observe an alteration of the *Pseudomonadota/Bacteroidota* (*P*/*B*) ratio (Figure 1D). In addition, *Verrucomicrobiota* was practically absent in the controls (0.1% vs. 4.56% *p* < 0.05; Figure 1C).

At the genus level, significant differences were found between the two groups (Figure 1E). The controls had higher abundances of *Bacteroides*, *Prevotella*, *Faecalibacterium*, *Blautia*, and *Agathobacter*, presenting certain genera not found in the controls like *Alloprevotella*, *Prevotellaceae UGC-001*, *Roseburia*, and *Parabacteroides*. The SLE group exclusive presented several other genera, including *Muribaculaceae*, *Enterococcus*, *Akkermansia*, *Asteroleplasma*, *Clostridia UCG-014*, *Dialister*, *Holdemanella*, and *Parasutterella UCG-002* (Figure 1E).

### 3.3. Identification of Specific Bacteria as Possible SLE Markers

**Figure 2:**
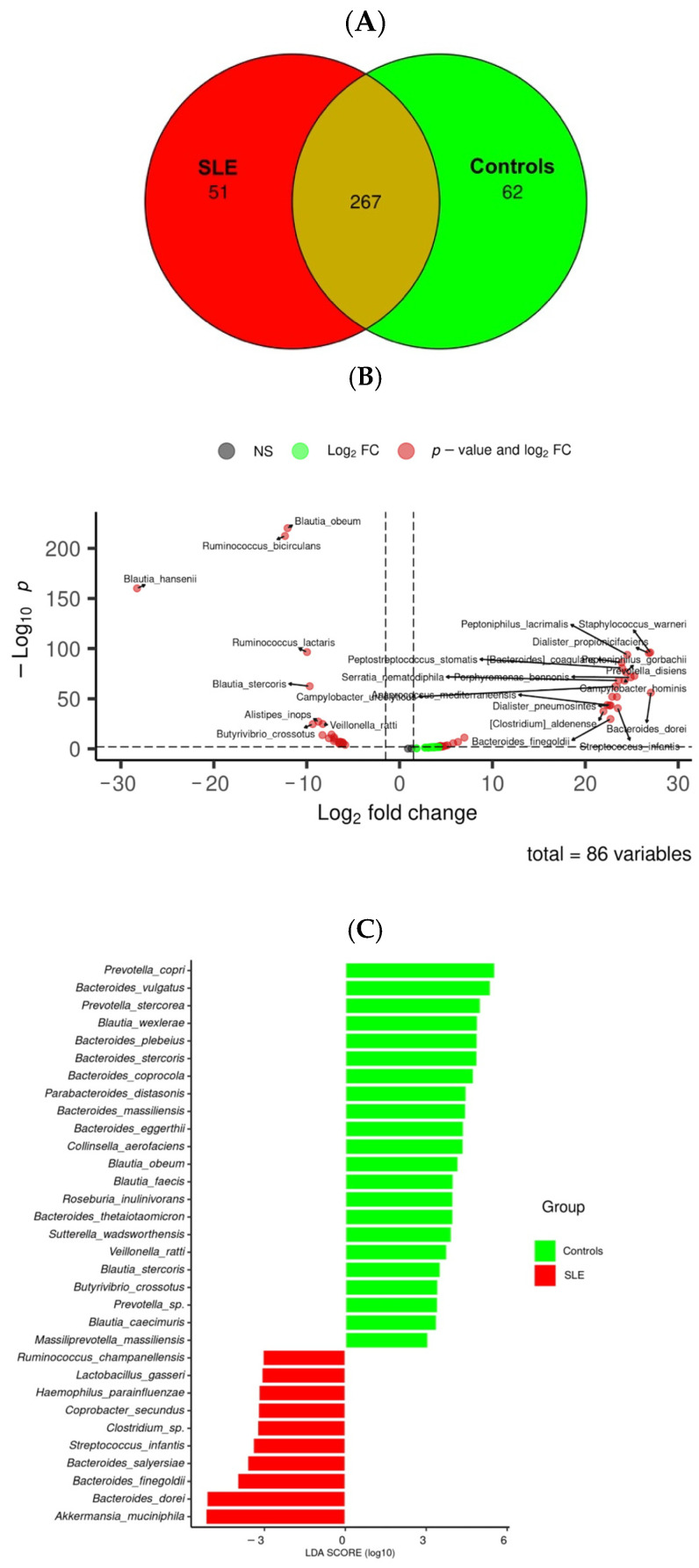
Gut microbiota diversity at species level in SLE patients and controls. SLE patients and controls presented differences in specific bacteria that could be used as possible biomarkers for systemic lupus erythematosus. (**A**) Venn diagram showing species distribution between control and SLE groups. (**B**) Volcano plot showing differential abundance taxa between SLE patients and controls. (**C**) Linear discriminant analysis effect size (LEfSe) showing the species presented in all faecal samples of each group.

To identify additional taxa that could be potential markers for SLE, we determined the core microbiota as well as the group-specific bacterial species (Figure 2A). A total of 267 species were shared between the two groups, highlighting the presence of a core microbiota that appears essential to the human gut ecosystem. The control group presented greater diversity, with 62 unique species, whereas the SLE group showed slightly reduced diversity, with 51 unique species.

Significant differences in microbial abundance were identified using a volcano plot, highlighting taxa that were enriched or depleted in the SLE patients compared to the controls (Figure 2B). In addition, core microbiome analysis using LEfSe (Linear Discriminant Analysis Effect Size) identified species consistently present in all faecal samples within each group (Figure 2C). Specifically, 22 taxa were detected in all the control samples, whereas only 10 taxa were found in all the SLE patients—namely *Akkermansia muciniphila*, *Bacteroides dorei*, *Bacteroides finegoldii*, *Bacteroides salyersiae*, *Streptococcus infantis*, *Clostridium* sp., *Coprobacter secundus*, *Haemophilus parainfluenzae*, *Lactobacillus gasseri*, and *Ruminococcus champanellensis*. This reduction in shared microbial taxa suggests a loss of microbial diversity associated with SLE.

### 3.4. Identification of Gut Microbiota Signatures Associated with Clinical Characteristics in SLE: A Machine Learning-Driven Discovery

To explore the association between clinical variables and gut microbiota in patients with SLE, an unsupervised machine learning approach was applied. The clustering process was predominantly driven by four variables: hypertension, corticosteroid therapy, menopausal status, and treatment with Belimumab (see Supplementary Figure S1). The resulting clusters were characterised as Cluster 1, consisting largely of premenopausal women receiving corticosteroids and/or Belimumab; Cluster 2, primarily composed of hypertensive individuals undergoing corticosteroid treatment with mixed menopausal status and lower Belimumab use; and Cluster 3, which included mostly menopausal patients with minimal or no exposure to corticosteroids or Belimumab and a lower prevalence of hypertension.

**Figure 3:**
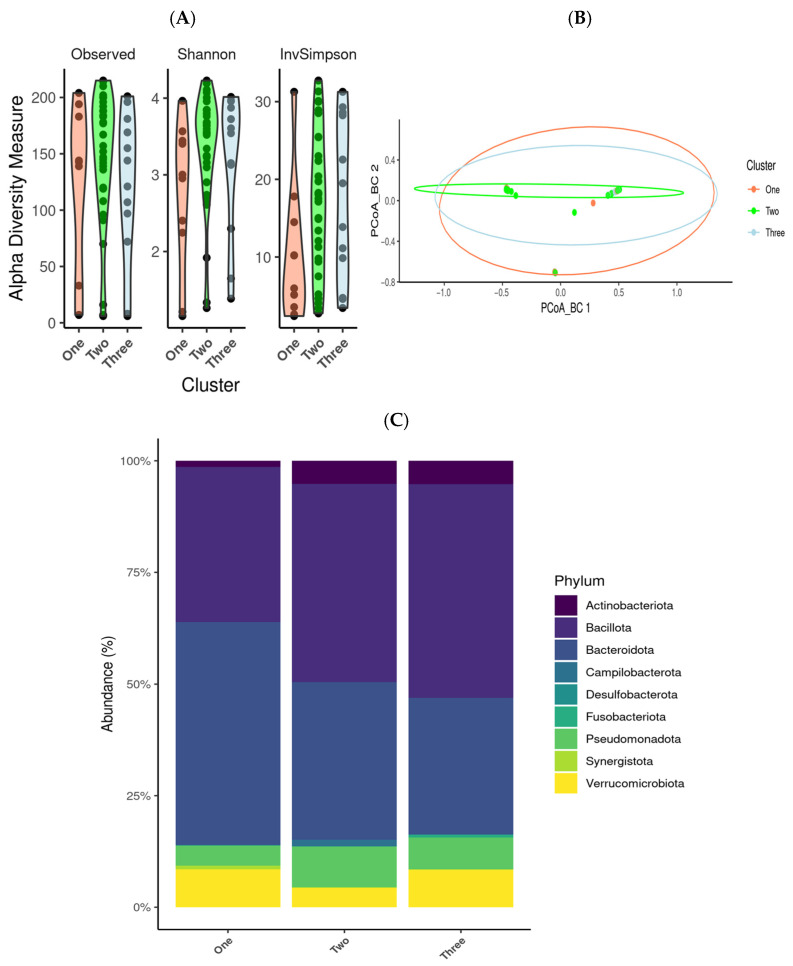

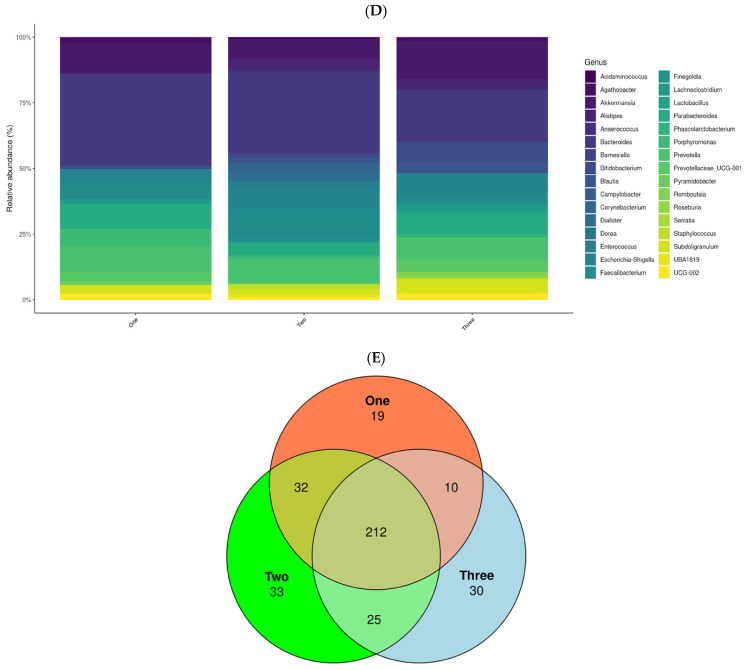
Analysis of gut microbiota associated with different clinical features (hypertension, menopause, corticosteroid treatment, and Belimumab treatment). SLE patients showed differences in gut microbiota diversity. (**A**) Alpha diversity (observed features, Shannon, and inverse Simpson) between Group 1, Group 2 and Group 3. (**B**) PCoA for Bray–Curtis diversity comparing groups. (**C**) Differences between groups at phylum level. (**D**) Genus abundance between Group 1, Group 2 and Group 3. (**E**) Venn diagram showing species distribution between SLE groups.

Alpha diversity metrics (Shannon, InvSimpson indexes and observed species) demonstrated no significant within-cluster differences (*p* > 0.05) (Figure 3A). Similarly, beta diversity analysis using the Bray–Curtis dissimilarity and PCoA showed no clear segregation between the clusters, a finding further supported by PERMANOVA (*p* = 0.05) (Figure 3B), suggesting that the overall microbial diversity (both within and between samples) was largely comparable across patient subgroups. Nevertheless, taxonomic profiling revealed significant shifts in phylum-level abundance. The most pronounced differences were observed in the phylum *Bacillota* and *Actinobacteria*, with a progressive increase in relative abundance from Cluster 1 to Cluster 3, whereas *Bacteroidota* displayed a notable decline across these groups (Figure 3C). At the genus level, significant variations were detected in several taxa, including *Bacteroides*, *Akkermansia*, *Bifidobacterium*, *Escherichia-Shigella*, *Faecalibacterium*, *Porphyromonas*, *Prevotella*, *Subdoligranulum*, *Alistipes*, *Blautia*, *Corynebacterium*, *Dialister*, and *Roseburia* (Figure 3D). These genera exhibited cluster-specific patterns, suggesting microbiota signatures reflecting underlying clinical characteristics.

Additionally, a Venn diagram analysis was conducted to identify microbial species uniquely associated with each cluster. While 212 species were common to all the groups, Group 1 had 19 exclusive species, Group 2 had 33, and Group 3 had 30. Notably, Groups 1 and 2 shared a further 32 species, suggesting a closer microbial relationship between these subgroups. (Figure 3E). In addition, microbial overlap was detected between Clusters 1 and 2, which shared the greatest number of species, while Clusters 1 and 3 and Clusters 2 and 3 shared only 25 and 27 species, respectively (Figure 3).

**Figure 4:**
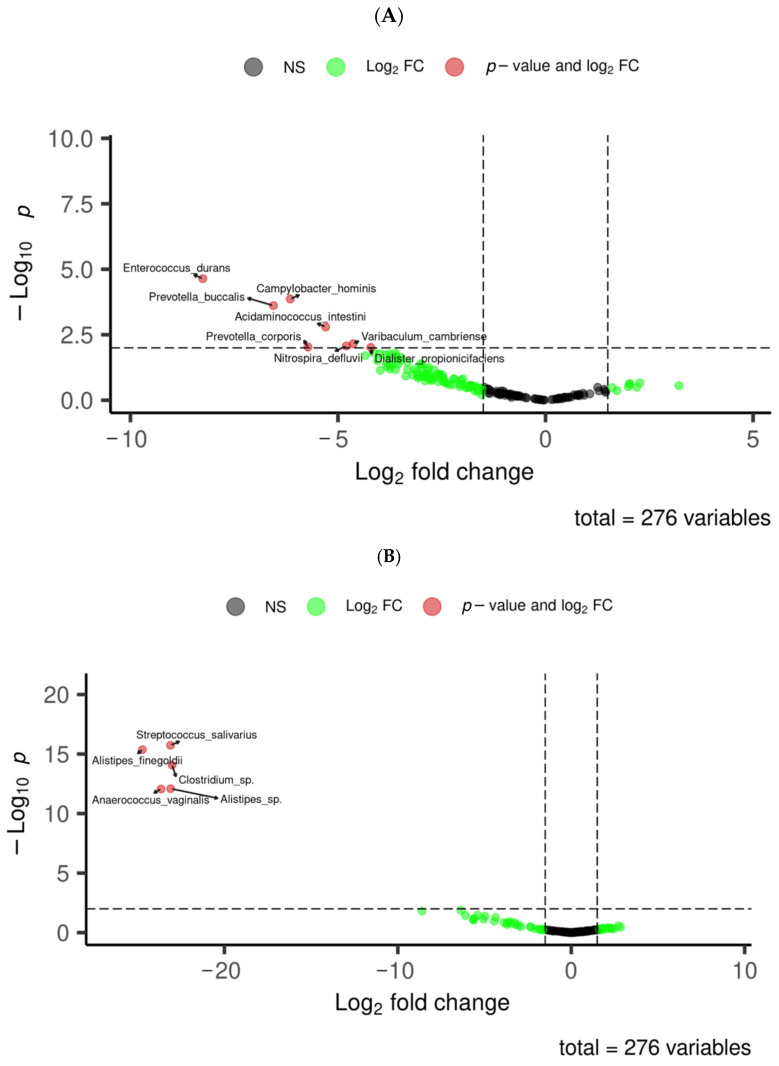

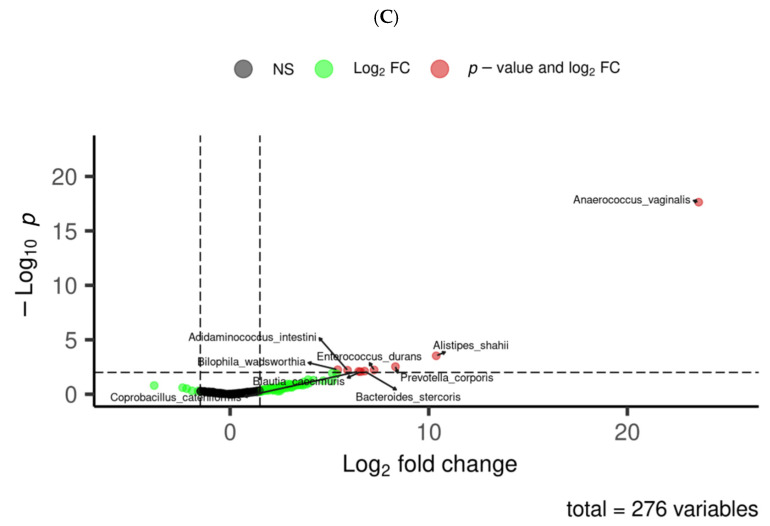
Differences in microbial profile between SLE patient groups (hypertension, corticosteroid treatment, and Belimumab treatment). Significant differences in microbial profile between SLE groups. (**A**) Volcano plot showing significant differences in hypertensive patients. (**B**) Volcano plot representing meaningful distinctions in the corticosteroid treatment group. (**C**) Volcano plot illustrating notable differences in the Belimumab treatment group.

We further investigated the specific clinical factors driving this variation. Firstly, we compared the gut microbiota between hypertensive and non-hypertensive SLE patients. Volcano plot analysis revealed a significant reduction in several bacterial species among hypertensive patients, including *Enterococcus durans*, *Campylobacter hominis*, *Prevotella buccalis*, *Acidaminococcus intestini*, *Prevotella corporis*, *Nitrospira defluvii*, *Dialister propionicifaciens*, and *Varibaculum cambriense* (*p* < 0.05) (Figure 4A). In addition, when considering corticosteroid treatment, we observed a significant depletion of key gut microbial taxa, including *Streptococcus salivarius*, *Alistipes finegoldii*, *Clostrifium* sp., *Anaerococcus vaginalis*, and *Alistipes* sp. (Figure 4B) in SLE patients. Conversely, patients receiving Belimumab presented a distinct microbial profile characterised by an enrichment of specific bacterial species such as *Alistipes shahii*, *Enterococcus durans*, *Bilophila wadsworthia*, *Acidaminococcus intestini*, *Prevotella corporis*, and *Bacteroides stercoris* (Figure 4C).

## 4. Discussion

The findings of this study provide further evidence supporting the role of gut microbiota alterations in SLE and its potential implications for disease pathogenesis and clinical manifestations.

Alpha diversity (a measure of richness and evenness) showed no significant difference between SLE patients and controls, like previous studies conducted on European cohorts [12,13]. A higher dispersion within the SLE group suggests increased microbial heterogeneity, which could reflect individual variations in disease progression, immune responses, or treatment regimens.

In contrast, beta diversity analysis revealed a clear separation between SLE patients and controls, highlighting significant structural differences in microbial community composition that could be attributed to SLE pathogenesis. These findings align with several previous studies reporting gut dysbiosis in SLE, where shifts in microbiota composition have been linked to SLE development, pathogenesis, and clinical manifestations [4,5].

At the phylum level, SLE patients showed increased levels of *Pseudomonadota*, resulting in an altered *P*/*B* ratio, consistent with previous reports [12,14]. This phylum’s expansion has also been associated with other autoimmune diseases such as inflammatory bowel disease (IBD) and rheumatoid arthritis (RA), where it is thought to influence immune homeostasis by modulating lymphocyte subpopulations and cytokine levels, potentially driving dysbiosis-related immune activation [13,14,15,16,17]. Consistent with earlier findings in SLE, we also observed a significant reduction in *Bacteroidota*, a phylum known for its role in the production of key microbial metabolites such as butyrate and propionate. These short-chain fatty acids (SCFAs) suppress the production of LPS-induced pro-inflammatory cytokines and modulate B cell activity by promoting the differentiation of extrathymic regulatory T cells (Tregs) [18]. Interestingly, *Bacteroidota* abundance has been shown to correlate with SLE disease activity index (SLEDAI) scores [5].

Conversely, there was a significant increase in the Verrucomicrobiota phylum (particularly the genus *Akkermansia*) in SLE patients. *Akkermansia* has been implicated in altered mucosal immune responses and has been linked to other autoimmune disorders, including multiple sclerosis (MS) and RA, suggesting a possible role in immune modulation [19,20,21].

Taken together, the enrichment of *Pseudomonadota* and *Verrucomicrobiota*, along with the depletion of *Bacteroidota*, may contribute to immune dysregulation in SLE through microbiota-driven activation.

At the genus level, there were notable shifts between SLE patients and controls in terms of microbial composition. The genera *Bacteroides*, *Prevotella*, *Faecalibacterium*, *Blautia*, and *Agathobacter* were more abundant in the controls, while *Alloprevotella*, *Roseburia*, and *Parabacteroides* were only found in this group. These genera are known for their anti-inflammatory properties and SCFA production, suggesting a protective role in maintaining gut immune balance [22,23,24]. In contrast, SLE patients exhibited an enrichment of potentially pathogenic genera, including *Muribaculaceae*, *Enterococcus*, *Akkermansia*, *Asteroleplasma*, *Clostridia UCG-014*, *Dialister*, *Holdemanella*, and *Parasutterella UCG-002*. This imbalance has previously been reported in SLE and other autoimmune diseases and may promote the pro-inflammatory environment characteristic of SLE [13,25,26]. Notably, 267 species were shared between the control and SLE groups, emphasising the stability of certain core microbiota essential for gut homeostasis despite the presence of the disease.

Another significant result was the identification of specific microbial signatures in different clinical subgroups of SLE patients, reinforcing the hypothesis that gut microbiota composition could contribute to disease heterogeneity. While no significant differences in alpha or beta diversity were found among the SLE groups stratified according to hypertension, corticosteroid, or Belimumab treatment, there were distinct phylum-level alterations. Specifically, *Bacillota* and *Actinobacteria* abundance increased progressively from Group 1 to Group 3, whereas *Bacteroidota* was markedly reduced. These shifts suggest a potential microbial gradient associated with clinical phenotypes, emphasising the role of gut microbiota in immune modulation and disease variability [27,28].

At the genus level, significant variations were detected between the three clinical subgroups, particularly in terms of *Bacteroides*, *Akkermansia*, *Bifidobacterium*, *Escherichia-Shigella*, *Faecalibacterium*, *Porphyromonas*, *Prevotella*, *Subdoligranulum*, *Alistipes*, *Blautia*, *Corynebacterium*, *Dialister*, and *Roseburia*. These genera displayed differential abundance patterns across groups, suggesting a potential microbial signature linked to specific clinical manifestations. Notably, *Akkermansia* and *Escherichia-Shigella*, previously associated with mucosal barrier dysfunction and systemic inflammation, were more abundant in Groups 2 and 3 [29,30]. Conversely, beneficial genera such as *Faecalibacterium*, known for anti-inflammatory properties and SCFA production, were reduced in Groups 1 and 3. Thus, impaired gut microbial homeostasis characterised by an increased abundance of pro-inflammatory genera together with the depletion of anti-inflammatory taxa may be related to different disease phenotypes [31,32,33].

Additionally, we observed a core microbiota of 212 species common to all the clinical groups, suggesting the presence of a shared microbial signature across all SLE patients regardless of clinical stratification. However, a substantial number of species exclusive to the SLE clinical subgroups were also identified, indicating potential microbial markers for distinct clinical subtypes. Specifically, Cluster 1 (premenopausal/immunomodulated) presented 19 unique species, perhaps reflecting the influence of hormonal status and immunomodulatory therapy on the gut ecosystem. Cluster 2 (hypertensive/moderate therapy) exhibited the highest number of unique taxa (33 species), suggesting that the combined effects of hypertension and corticosteroid use may shape a more distinct microbial environment. Cluster 3 (menopausal/low treatment) presented 30 exclusive species, potentially driven by lower therapeutic pressure and postmenopausal physiological changes. Notably, the degree of microbial overlap between the clusters revealed further insight into intergroup relationships. Clusters 1 and 2 shared the greatest number of species, reflecting a closer microbial resemblance, potentially influenced by similar treatment regimens (e.g., corticosteroids), despite a differing menopausal status. In contrast, Clusters 1 and 3 and Clusters 2 and 3 shared a smaller number of species, presenting greater divergence in microbial composition that could be attributed to treatment intensity and/or hormonal status.

Additionally, in relation to clinical manifestations and consistent with our findings, previous studies of SLE patients have reported cardiovascular complications, including hypertension, associated with higher levels of the phylum *Bacteroidota* and lower levels of the phylum *Bacillota* [34]. Although no prior studies have specifically examined the association between bacterial species and hypertension in SLE patients, our findings reveal a reduction in SCFA-producing species in the hypertensive subgroup, such as *Acidaminococcus intestini* and *Dialister propionicifaciens*, which may be linked to blood pressure regulation [35].

On the other hand, emerging evidence suggests that dysbiosis and microbiota metabolites (SCFAs and bile acids) are implicated in hypertension regulation [36]. Although preclinical and early clinical trials targeting gut microbiota are still limited, they show promising blood pressure modulation results. We report new insights in this area for SLE; however, future studies are needed to support our findings and characterise novel biomarkers and therapeutic targets of gut microbiota that could help prevent cardiovascular complications in SLE.

Similarly, our analysis of SLE patients treated with corticosteroids or Belimumab revealed significant shifts in gut microbial composition. These observations provide compelling evidence underscoring the capacity of different anti-inflammatory/immunomodulatory strategies to selectively shape the gut microbiome. Importantly, the emergence of potentially immunoregulatory taxa during Belimumab therapy suggests a novel mechanism through which biologics might act, not only by modulating immune cells, but also by reprogramming host–microbiota interactions, which are critical to disease pathogenesis and treatment response in SLE [37,38].

Taken together, our findings demonstrate distinct microbiota profiles across clinically stratified SLE groups and support the hypothesis that gut microbial signatures seem to be influenced not only by disease presence but also by specific clinical and therapeutic variables. Future research should focus on mechanistic studies to determine whether specific microbial alterations contribute to disease onset and progression or represent secondary changes due to SLE-associated inflammation and treatment effects. Longitudinal studies incorporating metagenomics, metabolomics, and host immune profiling will be essential to validate these findings and assess their potential for guiding microbiome-targeted therapies [39,40,41].

Some limitations should be acknowledged in this study. Firstly, its cross-sectional design limits the ability to infer causal relationships between gut microbiota alterations and the onset and progression of SLE. Longitudinal studies are necessary to determine whether the observed microbial changes precede the clinical symptoms or are secondary to the systemic inflammation, immune dysregulation, or therapeutic interventions. Secondly, although the stratification into clinical subgroups allowed for a more nuanced analysis, the sample size within each subgroup was relatively small, potentially limiting statistical power and generalisability. In addition, variables that can significantly impact gut microbiota composition such as dietary habits, smoking status, or prior antibiotic use were not fully controlled. Thus, larger, multi-centre studies that include lifestyle variables will be required to replicate these results in more diverse populations. Thirdly, this study relied on 16S rRNA sequencing, which, while effective for taxonomic profiling, lacks the resolution needed to identify strain-level variation and functional capacity. The integration of shotgun metagenomics, metabolomics, and transcriptomics would offer deeper insight into the metabolic and immunomodulatory roles of specific taxa in SLE.

## 5. Conclusions

In conclusion, our results reveal significant alterations in gut microbiota composition among SLE patients, with distinct profiles corresponding to different clinical subgroups. These findings support growing evidence that gut dysbiosis plays a role in SLE pathogenesis and highlights specific bacterial taxa that may serve as potential biomarkers for disease stratification. The enrichment of pro-inflammatory taxa and depletion of anti-inflammatory species suggest a microbiome imbalance that may contribute to immune dysregulation. Moreover, the identification of microbial signatures linked to clinical phenotypes underscores the potential for microbiota-based biomarkers in personalised SLE medicine.

Future functional and longitudinal studies incorporating host–microbiota interaction analyses will be essential in the development of microbiome-targeted therapies aimed at restoring gut homeostasis and improving clinical outcomes in SLE.

## Figures and Tables

**Table 1:** Main characteristics of the study groups.

	SLE (n = 57)	Controls (n = 57)	*p* Value
Age (years)	44.19 ± 11.88	43.5 ± 11.05	0.76
BMI	23.78 ± 4.75	24.13 ± 4.5	0.70
Clinical data			
Time of disease (years)	11.07 ± 8.87	NA	
SLEDAI-2K	5.81 ± 5.91	NA	
SLICC	1.05 ± 1.33	NA	
Antinuclear antibody quantification (U/mL)	9.47 ± 20.92	NA	
Anti-dsDNA quantification (U/mL)	58.80 ± 201.00	NA	
C3 complement (mg/dL)	99.24 ± 25.67	NA	
C4 complement (mg/dL)	19.38 ± 8.29	NA	
CRP (mg/dL)	2.40 ± 2.93	NA	
Comorbidities			
Diabetes (Yes)	3 (5.26%)	NA	
Hypertension (Yes)	16 (28.07%)	NA	
Cardiovascular disease (Yes)	6 (10.52%)	NA	
Renal disease (Yes)	10 (17.54%)	NA	
Arthritis (Yes)	14 (24.56%)	NA	
Osteopenia (Yes)	8 (14.03%)	NA	
Antiphospholipid syndrome (Yes)	2 (3.5%)	NA	
Sjögren syndrome (Yes)	10 (17.54%)	NA	
Raynaud’s syndrome (Yes)	7 (12.28%)	NA	
Rowell syndrome (Yes)	1 (1.75%)	NA	
Drug use			
Corticoids (Yes)	23 (40.35%)	NA	
Antimalarials (Yes)	50 (87.71%)	NA	
Azathioprine (Yes)	6 (10.52%)	NA	
Belimumab (Yes)	7 (12.28%)	NA	
Mycophenolate mofetil (Yes)	4 (7.01%)	NA	
Total drug use	3.12 ± 1.98	NA	

Note. The data is expressed as frequencies and percentages and as the mean and standard deviation (SD). Bold values signify a *p* value of less than 0.05. For control group (healthy subjects) clinical variables were not applicable (NA).

## Data Availability

Data is available on request from the authors.

## Supplementary Materials

The following supporting information can be downloaded at https://www.mdpi.com/article/10.3390/medsci13030151/s1: Figure S1: Categorisation of groups according to clinical parameters: hypertension, corticosteroid treatment, menopause, and Belimumab.
