# Supplementary material for: Association of Gut Dysbiosis with Disease Phenotype and Treatment in Systemic Lupus Erythematosus
Source: Med Sci (Basel). 2025 Aug 23;13(3):151. doi: 10.3390/medsci13030151 (PMC12452298; doi:10.3390/medsci13030151)
Supplement: Supplementary file 1 [file medsci-13-00151-s001.zip › medsci-3808443-supplementary.pdf]

medical sciences

MDPI

Supplementary material

Supplementary figure S1. Categorization of groups according to clinical parameters: hypertension, corticosteroid treatment, menopause and Belimumab

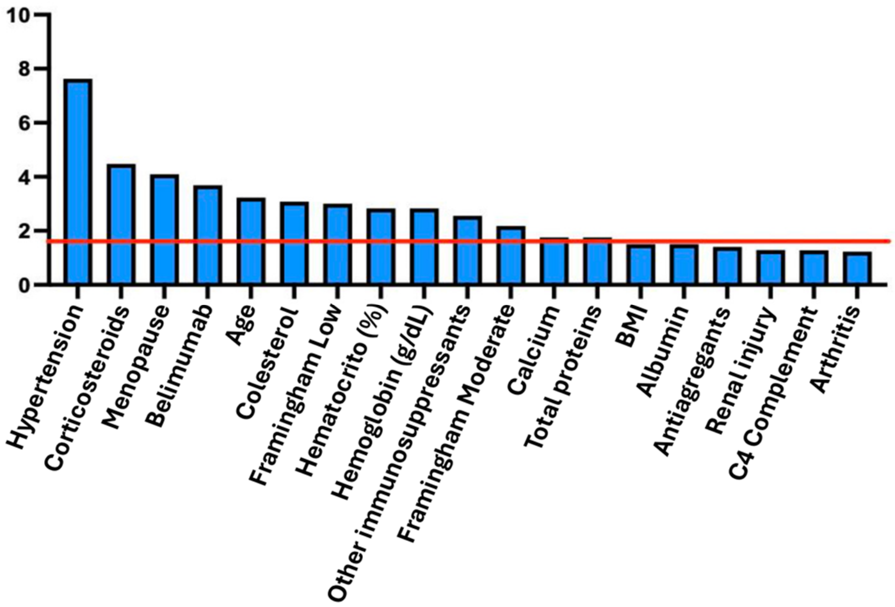

 https://doi.org/10.3390/xxxxx
